# Electron Microscopy Analysis of Hf–Induced Nanostructural Modifications in (Ti,Zr,Hf)NiSn Half-Heusler Thermoelectrics

**DOI:** 10.3390/nano15161250

**Published:** 2025-08-14

**Authors:** Isaak G. Vasileiadis, George P. Dimitrakopulos, Thomas Kehagias, Christina Karafyllia, Theodora Kyratsi, Andreas Delimitis

**Affiliations:** 1 Department of Physics, Aristotle University of Thessaloniki, 54124 Thessaloniki, Greece; isvasile@physics.auth.gr (I.G.V.); gdim@auth.gr (G.P.D.); kehagias@auth.gr (T.K.); ckarafyl@auth.gr (C.K.); 2 Department of Mechanical and Manufacturing Engineering, University of Cyprus, Nicosia 1678, Cyprus; kyratsi.theodora@ucy.ac.cy

**Keywords:** electron microscopy (TEM-HRTEM), electron diffraction, EDS, Geometrical Phase Analysis (GPA), half-Heusler thermoelectrics (TEs), energy harvesting

## Abstract

The structural features of Sb–doped (Ti,Zr)NiSn and (Ti,Zr,Hf)NiSn half-Heusler (HH) thermoelectrics have been identified down to the atomic scale using a combination of transmission electron microscopy (TEM) techniques. TEM sheds light on the morphology, phases present, size distributions and elemental variations between the two samples. Both materials consist of the HH phase, at both micro- and nanoscale levels, and comprise particles with two size ranges, 115 and 223 nm, on average, for large HH particles and 4–17 nm for nanoparticles for both materials. Hf incorporation in the HH lattice brought upon significant elemental fluctuations, manifested in chemical profiles and lattice parameter variations measured by post-experimental image analysis. The increased elemental variations induced by Hf substitution significantly contributed to the low thermal conductivity values and high power factor, leading to an enhanced figure of merit of 0.76 at 762 K for (Ti,Zr,Hf)NiSn, demonstrating the capability of TEM to confirm the structural features that are responsible for improved TE performance.

## 1. Introduction

The total amount of energy produced nowadays on a global scale that is lost as waste heat reaches up to a remarkable amount of around 66% [1]. Using this waste heat as a power source is tremendously beneficial. Thermoelectric (TE) devices comprise materials that can convert waste heat into useful electricity and vice versa, based on the Seebeck and Peltier effects. These devices can be rather reliable and compact as they do not contain moving parts and can be used in harsh environments, making them suitable for a variety of applications, including space ones [2].

TE efficiency is best defined by the figure of merit, which is equal to the following:

*zT* = *σS*^2^*T*/*κ* (1)

where *σ* is electrical conductivity; *S* is the Seebeck coefficient; *T* is the absolute temperature; and *κ* = *κ_Ι_* + *κ_e_* is the total thermal conductivity, comprising the lattice (*κ_Ι_*) and electronic (*κ_e_*) counterparts. Ways to increase *zT* include maximizing the Power Factor (PF), *σS*^2^, while minimizing thermal conductivity. Recent advances in this remedy focus on band reforming and nanostructuring approaches, such as the introduction of grain boundaries, point, line and planar defects and elemental alloying [3]. The ultimate goal is to considerably suppress *κ*, while preserving a high PF and, hence, a significantly high *zT*.

Full or half-Heusler (HH) materials have been widely known since the beginning of the 20th century due to their ferromagnetic behavior and, in recent years, for their promising spintronics applications [4]. In addition, they exhibit promising TE properties for high temperature applications due to their enhanced power factor values and prolonged stability at higher temperatures [5]. However, they suffer from relatively high thermal conductivity [6], which lowers their performance [7]. Ways to accommodate this include alloying and nanostructuring approaches [6], with a view to increase phonon scattering through alloy elements fluctuations, imposed strain and the creation of a significant density of grain boundaries.

Among the various n-type HHs, ZrNiSn or HfNiSn and their doped counterparts exhibit improved TE performance as a result of their nanostructural features. Studies by Chauhan et al. [3] and Du et al. [8] pointed out the necessity of introducing lattice point defects, such as interstitials or substitutional ions, as an effective way of scattering short-wavelength phonons and hence reducing lattice thermal conductivity. However, the exact nature, type, locations and distribution of such defect centers are yet to be clarified to effectively design n-type HHs with enhanced TE properties, such as low thermal conductivity, and a high Seebeck coefficient, power factor and *zT*.

Recent structural analyses employing electron microscopy are focused on systems for renewable technologies and energy harvesting, with TE materials being among them [9]. Transmission electron microscopy (TEM) and associated techniques have become indispensable tools for unraveling the intricate nanostructure of HH materials, particularly those based on the multicomponent Ti-Zr-Hf-Ni-Sn-Sb system. These complex alloys exhibit a rich microstructure influenced by multi-element substitution, which can significantly impact thermoelectric performance. In a study by Morimura et al. [10], high-resolution TEM (HRTEM) was employed to investigate the structural features of Ti_0.5_(Zr_0.5_Hf_0.5_)_0.5_NiSn_0.998_Sb_0.002_ alloys. The authors observed nanoscale domain structures, typically ranging from 2 to 10 nm in size, that revealed varying degrees of atomic ordering. Fourier and inverse Fourier transformation analyses confirmed the coexistence of both ordered and disordered phases, particularly in the second nearest-neighbor positions, suggesting a complex local atomic arrangement that contributes to phonon scattering and affects thermal conductivity. In another investigation, Populoh et al. [11] examined multiphase Ti-Zr-Hf-Ni-Sn systems and reported phase separation into Ti–rich and Zr/Hf–rich domains at a nanometer scale. TEM observations confirmed the coexistence of different half-Heusler phases while energy dispersive X-ray spectroscopy (EDS) revealed that Ni and Sn remained uniformly distributed, whereas the elemental segregation of Ti, Zr and Hf led to compositionally distinct nanodomains. This phase separation was linked to enhanced phonon scattering and improved thermoelectric performance, with *zT* values reaching up to 1.0 in a temperature range from 675 to 775 K.

The nanostructural features of binary and ternary HH materials, of the general type (Ti_0.4_Zr_0.6_)_1−x_Hf_x_NiSn (x = 0, 0.3), are dealt with in a comparative manner in the current study. The selected HH materials, doped with low amounts of Sb (1.5 and 2 at%, respectively), were synthesized by an easy, inexpensive and timely one-step mechanical alloying method [12,13], and they have shown a remarkable improvement in their TE properties, especially power factor, electrical conductivity and resulting figure of merit *zT*. Variations in particle size, morphological characteristics, phase multiplicity, crystalline quality and lattice constant variations have been assessed by a combination of TEM/HRTEM, selected area diffraction (SAD), EDS and post-experimental HRTEM image analysis employing Geometrical Phase Analysis (GPA) [14]. Structural features mainly responsible for TE property enhancement form the basis for synthesizing tailor-made TE nanomaterials with considerable alloying, i.e., high entropy TEs, while preserving optimum TE performance by not sacrificing mobility, thermal or electrical conductivities.

## 2. Materials and Methods

High-purity elemental powders of Ti (99.99% Alfa Johnson Matthey GmbH, Sulzbach, Germany), Hf (99.6% Alfa Johnson Matthey GmbH, Sulzbach, Germany), Zr (99% US Research Nanomaterials Inc., Houston, TX, USA), Ni (99.99% Sigma Aldrich Merck, Darmstadt, Germany), Sn (99.85% Alfa Johnson Matthey GmbH, Sulzbach, Germany) and Sb (99.9% Alfa Johnson Matthey GmbH, Sulzbach, Germany) were weighed according to the selected compositions (Sb–doped Ti_0.4_Zr_0.6_NiSn_1−x_Sb_x_ with x = 0.01, 0.015 and 0.02 or (Ti_0.4_Zr_0.6_)_0.7_Hf_0.3_NiSn_1−x_Sb_x_ with x = 0.01, 0.015, 0.02 and 0.025) in a tungsten carbide ball milling vial with a ball-to-material ratio of 10:1 as described in more detail in [12,13].

Due to the most promising TE property results and the degree of alloying, Hf–free samples (binary) containing 1.5 at% Sb (Ti_0.4_Zr_0.6_NiSn_0.985_Sb_0.015_) and Hf–rich samples (ternary) containing 2 at% Sb [(Ti_0.4_Zr_0.6_)_0.7_Hf_0.3_NiSn_0.98_Sb_0.02_] were selected for dedicated TEM/HRTEM observations and post-experimental analysis. Samples for TEM analysis were prepared by sonication of finely crushed material in high-purity ethanol, followed by drop casting the solution on ultrathin lacey C-films supported on 3.05 mm copper grids (Agar Scientific Ltd., Essex, UK). TEM experiments were carried out in JEOL 2100 HT and JEOL 2010 UHR electron microscopes (JEOL Ltd., Tokyo, Japan), operating at 200 kV, with point resolutions of 0.25 and 0.194, respectively. EDS measurements were performed with the JEOL 2100 HT electron microscope, fitted with an EDAX Apollo XLT silicon drift EDS detector (EDAX, Pleasanton, CA, USA), with an ultra-thin window and an energy resolution of 129 eV (Mn-K_a_ line). Spectrum acquisition and analysis were performed using TEAM EDS Suite v2.0 software.

## 3. Results and Discussion

HH compounds crystallize in the MgAgAs-type structure at the high symmetry F$\bar{4}$3m space group (#216), comprising three interpenetrating fcc sublattices. In principle, in an ideal unit cell, Sn ions occupy the octahedral 4a (0, 0, 0) sites (C sites) and Ti/Zr/Hf ions occupy the 4b (½, ½, ½) sites (N sites), whereas Ni ions occupy one of the two tetrahedral 4c sites, (¼, ¼, ¼) [or, equivalent (¼, ¼, ¾)] (T sites) [15].

Mechanical alloying, followed by hot press sintering of the samples resulted in the formation of a single HH phase, both for the binary (Ti-Zr) and ternary (Ti-Hf-Zr) materials. This has been previously identified by X-ray diffraction [12,13], where the formed HH phase in each case exhibited narrow XRD peaks, after a minimum milling time of 7 h at a speed of 600 rpm. In addition, the binary Ti_0.4_Zr_0.6_NiSn_0.985_Sb_0.015_ sample showed the presence of a Ni_3_Sn_4_ phase, even after hot pressing; such impurities are a common feature in this system. Interestingly, this impurity did not exist in the Hf–containing samples.

**Figure 1:**
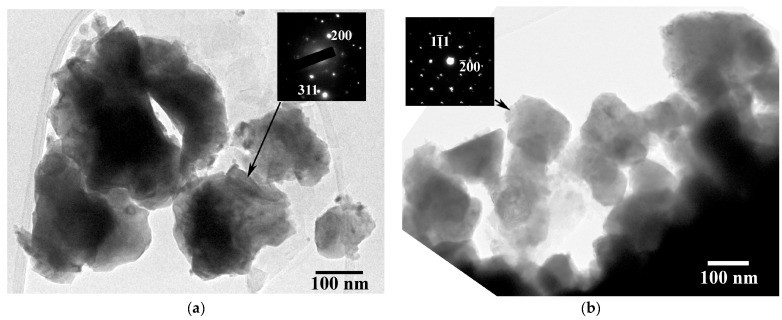
TEM images from (**a**) Ti_0.4_Zr_0.6_NiSn_0.85_Sb_0.015_ and (**b**) (Ti_0.4_Zr_0.6_)_0.7_Hf_0.3_NiSn_0.98_Sb_0.02_ HH materials, along with their corresponding SAD patterns (insets). The black arrows point to the particles that the SAD patterns were acquired from in each sample.

The morphology and particle size distributions of the HH phase in the samples are depicted in the TEM images of Figure 1. The HH particles appear agglomerated, with no specific shape and have an average size of 115 nm in Ti_0.4_Zr_0.6_NiSn_0.85_Sb_0.015_ and 223 nm in (Ti_0.4_Zr_0.6_)_0.7_Hf_0.3_NiSn_0.98_Sb_0.02_. Their size distributions range from 60 to 210 nm and 95 to 405 nm, respectively; hence, Hf incorporation brought upon a significant increase in HH particle sizes. Nevertheless, the average grain size of our samples remains low, compared to the ~300 nm reported so far [16]. The corresponding SAD patterns in Figure 1a,b reveal that they are single crystalline materials; the [011] zone axis pattern of Figure 1b particularly supports this. Measurements of the reciprocal lattice spacings assigned them to mixed HH phases; the results are listed in Table 1.

The theoretical values of the lattice constants were calculated from the HH end members TiNiSn (*a_TiNiSn_* = 0.594 nm), ZrNiSn (*a_ZrNiSn_* = 0.612 nm) and HfNiSn (*a_HfNiSn_* = 0.609 nm) using Vegard’s law. The experimental values, both from XRD [12,13] and SAD, comply well with the theoretical ones. In addition, Zr and/or Hf substitution for Ti in the binary or ternary HH compounds resulted in an increase in the lattice constant; the TiNiSn end member possesses by far the smallest lattice constant among the three of them.

**Figure 2:**
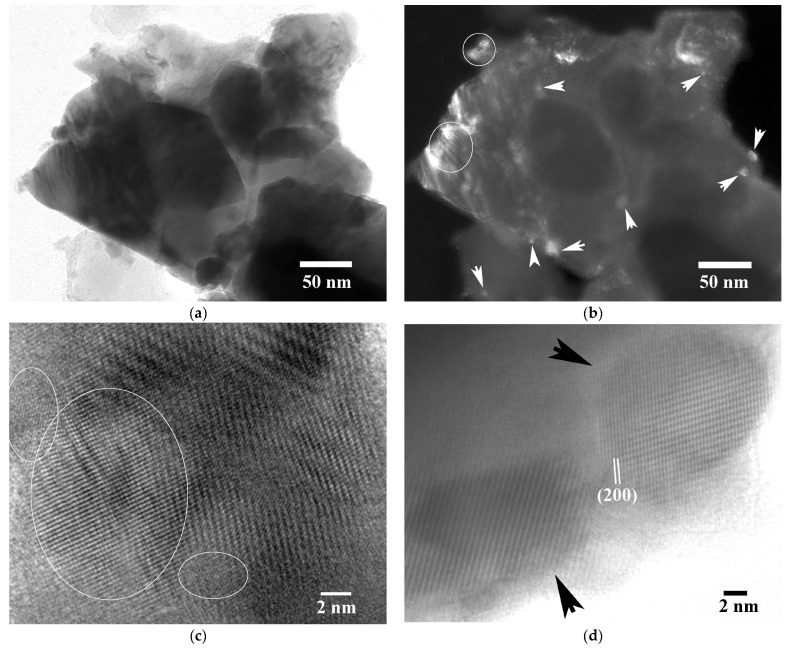
(**a**,**b**) Complementary BF and DF images of the (Ti_0.4_Zr_0.6_)_0.7_Hf_0.3_NiSn_0.98_Sb_0.02_ sample, where nanograin morphology is shown by white arrows in the DF image. The Moiré fringe patterns formed at areas within the white circles in the same image (DF) denote crystalline particle superposition. (**c**,**d**) HRTEM images of nanoparticles from Ti_0.4_Zr_0.6_NiSn_0.985_Sb_0.015_ and (Ti_0.4_Zr_0.6_)_0.7_Hf_0.3_NiSn_0.98_Sb_0.02_, respectively. The white-circled nanoparticles in (**c**) confirm their agglomeration, whereas the discrete nanoparticles in (**d**) are shown by black arrows.

As a consequence of ball milling synthesis, the samples contain a significant number of nanoparticles and nanograins, agglomerated with larger particles. Nanograin morphology is illustrated in the two complementary bright- (BF) and dark-field (DF) images of Figure 2a,b, respectively, from the ternary (Hf–containing) sample. The nanograins are clearly distinguished in the DF image. Along with the large particles, they also exhibit high crystallinity, as indicated by the Moiré fringe patterns created in several areas of the BF/DF images (circled in the DF image for clarity), as a consequence of their superposition on top of them or on the large particles. The coexistence of numerous large and, especially, small nanoparticles in patchy morphology and dense agglomeration in the HH samples typically results in the creation of a large proportion of grain boundaries that effectively act as scattering centers for phonons, leading thus to a reduction in thermal conductivity, as already manifested, initiated from binary Ti_0.4_Zr_0.6_NiSn_0.85_Sb_0.015_.

Nanoparticle formation has been best proven by HRTEM experiments. Figure 2c presents agglomerated nanoparticles in Ti_0.4_Zr_0.6_NiSn_0.85_Sb_0.015_ whereas a discrete morphology is depicted in Figure 2d from (Ti_0.4_Zr_0.6_)_0.7_Hf_0.3_NiSn_0.98_Sb_0.02_. Measurements of their size distributions from HRTEM images resulted in 5–15 nm for Ti_0.4_Zr_0.6_NiSn_0.85_Sb_0.015_ and 4–17 nm for (Ti_0.4_Zr_0.6_)_0.7_Hf_0.3_NiSn_0.98_Sb_0.02_. Although a greater size and wider distribution has been measured for the large particles in ternary HH, both samples exhibited similar ranges and sizes for their small nanoparticles. This can be attributed to ball milling synthesis of both materials, which produces particles with similar size distributions at the nanoscale level. The high percentage of small nanoparticles in ternary (Ti_0.4_Zr_0.6_)_0.7_Hf_0.3_NiSn_0.98_Sb_0.02_ compensated for the greater size of the large particles in it. The nanoparticles in both HRTEM images mainly exhibit (200) lattice fringes, whereas the single crystalline character is also manifested by the Moiré fringes there. Detailed measurements of the lattice fringe spacings in similar HRTEM images of both samples have been performed in order to derive the ‘nanometer’ scale lattice constant (*a_HRTEM_*), and the results are included in Table 1. There, it is shown that the average lattice constants in the nanoscale are in good agreement with the theoretical ones for both samples. However, it should be noted that the *a_HRTEM_* constant range for the Hf–containing sample was a bit greater, ranging from 0.587 nm to 0.628 nm, taking into account an error of 0.5% in the measurements. This indicates that, although the experimentally measured average lattice constant of the HH nanoparticles appears to approach the theoretical one, there are certain deviations, which imply elemental fluctuations, point defects, interstitials and atomic substitutions in the mixed HH phases. This is in very good agreement with an EXAFS analysis previously published for samples belonging to the same family of HH materials [15].

**Figure 3:**
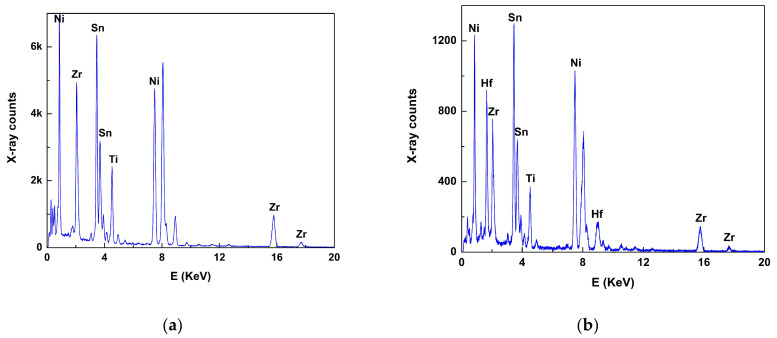
EDS spectra from representative particles in the two HH samples, (**a**) binary (Ti_0.4_Zr_0.6_NiSn_0.985_Sb_0.015_) and (**b**) ternary [(Ti_0.4_Zr_0.6_)_0.7_Hf_0.3_NiSn_0.98_Sb_0.02_].

Energy dispersive X-ray spectroscopy (EDS) confirmed the aforementioned findings. Figure 3 shows two representative EDS spectra from the binary and ternary HH materials. The spectra show all element peaks of the compounds, as well as the dopant (Sb). The latter, however, was difficult to distinguish due to its low content (1.5 at% and 2 at%, respectively) and overlap with multiple Sn L-family lines; therefore, the combination of these two elements is considered in any quantitative calculations from now on. EDS spectra did not reveal any other impurities; interestingly, O content was also negligible in the particles, which illustrates the chemical stability of the ball-milled TE materials [12].

**Figure 4:**
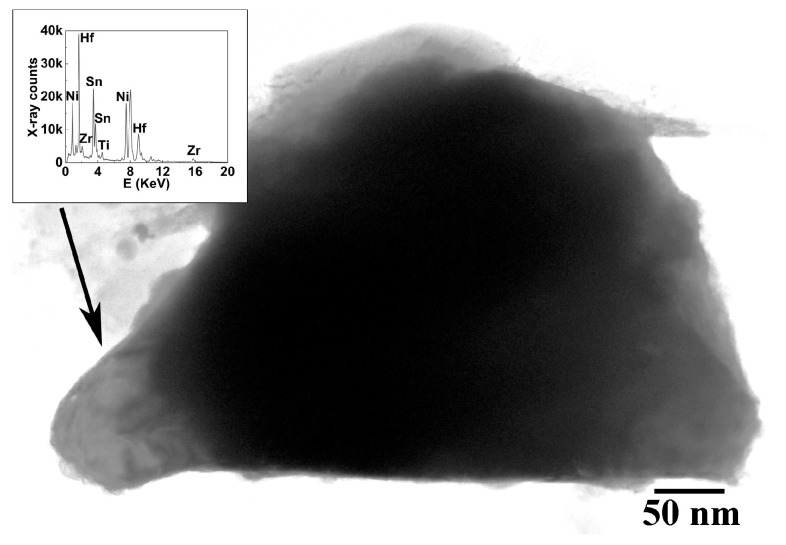
TEM image of a large particle with a Hf–rich domain (black arrowed) in ternary (Ti_0.4_Zr_0.6_)_0.7_Hf_0.3_NiSn_0.98_Sb_0.02_, along with its corresponding EDS spectrum inset.

Quantitative analysis showed that, on average, the elemental ratios are in good agreement with their theoretical contents for both materials, taking into account an EDS accuracy of 3–5% and provided that the sums of the elements in their nominal sites (Ti + Zr + Hf) and (Sn + Sb) are predominately considered. However, there are elemental variations among distinct particles, which tend to be more pronounced in the ternary (Hf–containing) sample. The results are presented in Table 2 in more detail and show that the elemental variations are more pronounced for Zr and Hf contents in particular, which implies elemental fluctuations among the HH particles down to the nanometer scale. This is in line with other studies [11] where Ti–, Hf– and Zr–based nanoscale domains co-existed within a HH matrix, with a positive effect on TE performance. On the other hand, Ti content tends to be more stable in our materials. To further support this interesting finding, regions in (Ti_0.4_Zr_0.6_)_0.7_Hf_0.3_NiSn_0.98_Sb_0.02_ have been detected with increased Hf content. Figure 4 confirms the latter case, where a particle with an increased Hf content domain is shown, along with its EDS spectrum inset. Hf content in these particle regions is three times higher than the nominal content, whereas there are greater deficiencies in Zr and Ti contents (−64 and −51 at%, respectively) compared to Ni and (Sn + Sb) ones; −16 and −12 at%, respectively, for these two crystallographic site elements. This reveals that Hf predominately substitutes for its corresponding N site elements in such Hf–rich particles [15]. Having clarified this, any remaining Hf content was found in either Hf–rich particle locations or, potentially, other crystallographic sites in the HH lattice. This is in line with TE property measurements [13], where a decrease in the Seebeck coefficient and an increase in carrier concentration have been reported as a consequence of Hf incorporation. Furthermore, as the substitution of Zr by Hf is isoelectronic, the increase in carriers has been attributed to defect creation (vacancies and/or interstitials), which is confirmed by ion substitution, vacancies or interstitial phenomena (i.e., alloy fluctuation) as implied by SAD/HRTEM and shown by EDS.

It should be clarified that the Hf–rich particle in Figure 4 represents a minority feature of the sample, merely identified due to the resolving power of TEM. As a more representative outcome, less Hf–rich regions have been sometimes encountered, where Hf excess was about twice as high, whereas Zr, Ti, Ni and (Sn + Sb) deficiencies reached −15, +1 (i.e., excess), −13 and −9 at%, respectively. This shows that Hf incorporation in the ternary HH has a diverse substitutional behavior, ranging from Hf–rich domains in discrete particles with excessive substitution for Zr and Ti, to particles with concentrations closer to the nominal case. In both cases, however, certain deviations from stoichiometry for the other crystallographic site elements (Sn/Sb for C sites and Ni for T sites) are frequently observed to the same extent (10–15 at%). Beyond this, any variations in Sn content in both doped sample categories may be attributed to the peak overlapping with Sb, which shows a very high content for the same reason. The total (Sn + Sb) content is more accurate in this case, which indeed shows a small difference compared with the nominal value.

**Figure 5:**
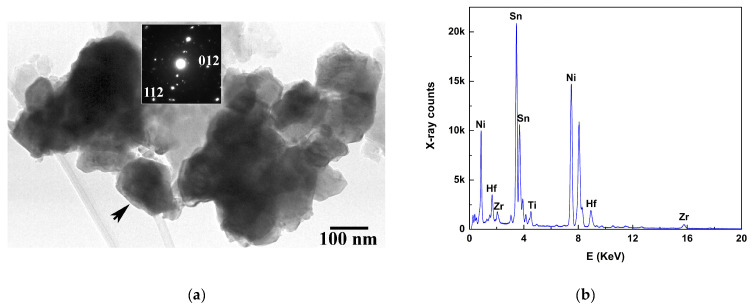
(**a**) Ni_3_Sn_4_ particle (black-arrowed) in the binary Ti_0.4_Zr_0.6_NiSn_0.985_Sb_0.015_ HH. The main reflections in the pattern are indexed to the Ni_3_Sn_4_ phase. (**b**) EDS spectrum from this particle, further confirming the Ni_3_Sn_4_ formation.

TEM characterization also sheds light on the existence of additional minority phases often encountered in HH TEs [12]. In particular, the presence of discrete Ni_3_Sn_4_ particles has been confirmed by SAD and EDS measurements only in the binary HH sample. This distinct morphology is best presented in Figure 5a, where the black-arrowed Ni_3_Sn_4_ particle has a size of ~120 nm and is crystalline as proven by its SAD pattern (inset). The EDS spectrum in Figure 5b further confirms this, whereby the Ni and Sn peaks are far more prominent in relation to the Zr, Ti and Hf ones, the latter three stemming from overlapping HH particles. Taking into account that TEM can easily identify discrete phases at the nanoscale, it has to be stated that such phases were seldom encountered in our materials; the absence of such additional phase formation in (Ti_0.4_Zr_0.6_)_0.7_Hf_0.3_NiSn_0.98_Sb_0.02_ further proves this.

**Figure 6:**
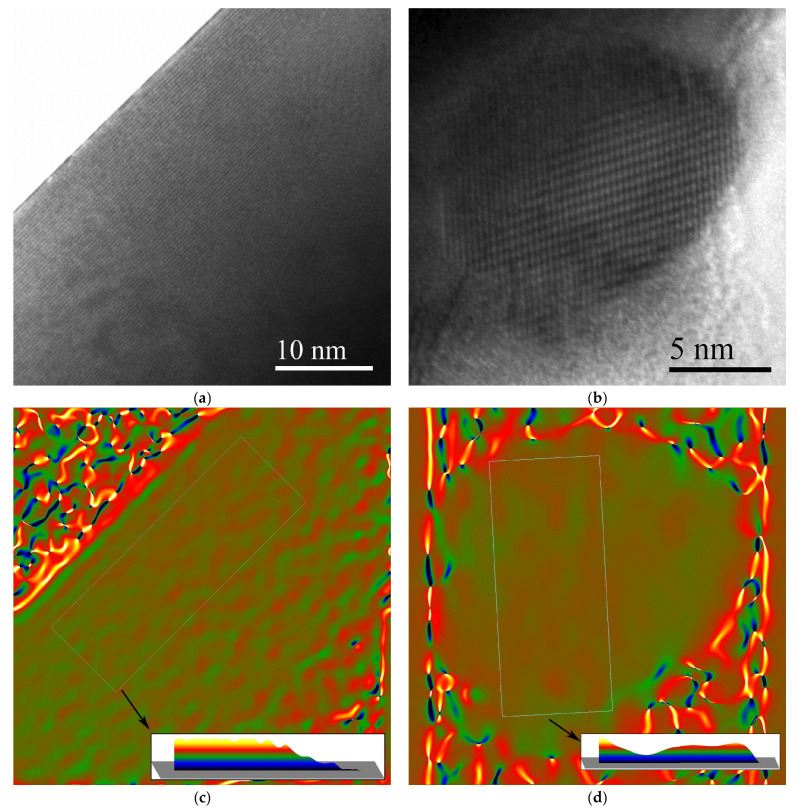
HRTEM images and GPA analysis from areas of the ternary HH. (**a**) HRTEM image from a large particle and (**c**) respective GPA map of the reduced relative variation in the lattice constant obtained using the ***g_111_*** spatial frequency. (**b**) HRTEM image of a nanoparticle and (**d**) respective GPA map using ***g_200_*** showing a uniform distribution of the lattice constant. The insets in (**c**,**d**) give the profiles of the variation along the indicated areas in the GPA maps.

Lattice parameter variations can affect TE properties, thermal conductivity in particular [17,18]. These may stem from deviations from the nominal chemical composition, attributed to substitution ions, point defects or interstitials. In order to assess this contribution, the lattice constant was probed by Geometrical Phase Analysis (GPA) [14] on HRTEM images, and characteristic results are presented in Figure 6 for both large and small particles in the ternary (Hf–containing) material. GPA analysis from a large (Ti_0.4_Zr_0.6_)_0.7_Hf_0.3_NiSn_0.98_Sb_0.02_ particle (Figure 6a,c) using the ***g_111_*** spatial frequency reveals a gradual transition of the lattice constant reaching up to 0.8% along the area indicated in Figure 6c (spatial resolution 1.4 nm and standard deviation 0.5%). On the other hand, in smaller areas such variations are not discernible as shown for a small (Ti_0.4_Zr_0.6_)_0.7_Hf_0.3_NiSn_0.98_Sb_0.02_ nanoparticle presented in Figure 6b and the respective GPA analysis in Figure 6d, showing variation in the ***g_200_*** spatial frequency. A uniform distribution of the lattice constant is demonstrated with a 0.4% standard deviation using 1.2 nm spatial resolution. Overall Hf incorporation in the matrix may result in lattice constant variations over larger areas due to elemental fluctuations as evidenced by the TEM methods used in this study.

The nanostructural features revealed by the combined TEM methods had a significant influence on TE properties; the latter were presented in previously published works [12,13]. There, both Ti_0.4_Zr_0.6_NiSn_0.85_Sb_0.015_ and (Ti_0.4_Zr_0.6_)_0.7_Hf_0.3_NiSn_0.98_Sb_0.02_ showed lower thermal conductivity values; prior to Sb doping, −2.63 W/mK, and 2.1 W/mK at room temperature, respectively. Such values are lower, in general, than the ones reported for samples synthesized by arc melting [2]. The reduced particle sizes along with the considerable elemental fluctuations and resulting lattice constant variations revealed in this TEM study resulted in such low *κ* values. Even for the ternary, Hf–containing sample, the larger particle size observed by conventional TEM imaging was compensated for by the increased elemental fluctuations caused by Hf incorporation as deduced by SAD, HRTEM and EDS. In addition, the latter sample showed a significant amount of nanostructuring, preserving the low range of size values, as in Ti_0.4_Zr_0.6_NiSn_0.85_Sb_0.015_. Concerning electrical properties, both the binary and ternary samples exhibited (prior to Sb doping) a high (absolute) Seebeck coefficient of about −205 μV/K and very promising power factors in the range of 22–26 μW/cmK^2^ at 300 K; in addition, the ternary, Hf–containing sample preserved a good electrical conductivity of ~220 S/cm, comparable to 205 S/cm for the binary sample. Despite the fact that Hf substitution for Zr is isoelectronic, the increased carrier concentration that led to high *S* and *σ* [19] can be attributed to a significant density of Hf–driven defects that act as donors. This is in full agreement with Hf–induced alloy fluctuations identified by the TEM analysis. Sb doping predominately brought upon a significant increase in electrical conductivity in both samples, −1681 S/cm and 2250 S/cm at 300 K for the binary and ternary samples, respectively, revealing a donor concentration increase by the dopant. This led to a significant increase in power factors, having a maxima of 32 μW/cmK^2^ for Ti_0.4_Zr_0.6_NiSn_0.85_Sb_0.015_ and 38 μW/cmK^2^ for (Ti_0.4_Zr_0.6_)_0.7_Hf_0.3_NiSn_0.98_Sb_0.02_ at 300 K. The increased power factors and relatively low thermal conductivities of the samples resulted in high *zT* values of 0.71 and 0.76 at 800 K and 762 K, respectively.

## 4. Conclusions

The structural characteristics of improved performance n-type HH (Ti,Zr,Hf)NiSn TE nanomaterials synthesized by one-step ball milling and doped with Sb have been determined by a combination of TEM methods. Both materials almost exclusively consist of a single HH phase, down to the nanoscale, being (Ti,Zr)NiSn and (Ti,Zr,Hf)NiSn, respectively. Their smaller particle sizes compared to previously reported similar compounds effectively contributed to their thermal conductivity reduction. Hf incorporation brings upon an increase in particle size in the ternary material compared to the binary one and an absence of additional discrete phases commonly encountered in HHs, such as Ni_3_Sn_4_, stabilizing the exclusive presence of the sole HH phase. At the same time, a significant elemental fluctuation has been identified by SAD, HRTEM and EDS analyses, attributed mainly to Hf incorporation, as the binary (TiZr)NiSn material shows smaller elemental variations. The observed elemental fluctuations and lattice parameter variations, predominately in the Hf–containing sample, suggest the introduction of point structural defects and local substitutional phenomena. These significantly influence carrier concentration and phonon scattering, which contribute to electrical conductivity, power factor enhancements and thermal conductivity reduction; hence, alloying provides a good background for further improvements by Sb doping. This shows that the ball milling of complex, multi-element alloy HH single phase TEs is feasible, maintaining a high TE performance and resulting in the highest figure of merit of 0.76 at 762 K for (Ti_0.4_Zr_0.6_)_0.7_Hf_0.3_NiSn_0.98_Sb_0.02_.

## Figures and Tables

**Table 1:** Measurements of the lattice constant in the Ti_0.4_Zr_0.6_NiSn_0.85_Sb_0.015_ (binary) and (Ti_0.4_Zr_0.6_)_0.7_Hf_0.3_NiSn_0.98_Sb_0.02_ (ternary) samples as derived from the SAD pattern insets of Figure 1.

Lattice Constant (nm)	Binary Ti_0.4_Zr_0.6_NiSn_0.985_Sb_0.015_	Ternary (Ti_0.4_Zr_0.6_)_0.7_Hf_0.3_NiSn_0.98_Sb_0.02_
*a_theor_*	0.605	0.606
*a_XRD_*	0.602 (±0.002)	0.604 (±0.002)
*a_SAD_*	0.601 (±0.002)	0.605 (±0.002)
*a_HRTEM_*	0.606 (±0.006)	0.604 (±0.008)

**Table 2:** EDS analysis results from the two n-type HH samples.

	Ti K (at%)	Zr L (at%)	Hf M (at%)	Ti + Zr + Hf (at%)	Ni K (at%)	Sn L (at%)	Sb L (at%)	Sn + Sb (at%)
**Ti_0.4_Zr_0.6_NiSn_0.985_Sb_0.015_**	13.5	18.7	-	32.2	33.6	31.5	2.5	34
Nominal	13.3	20	-	33.3	33.4	32.8	0.5	33.3
**(Ti_0.4_Zr_0.6_)_0.7_Hf_0.3_NiSn_0.98_Sb_0.02_**	9.6	10.4	13.8	33.8	34	28	4	32
Nominal	9.33	14	10	33.3	33.4	32.7	0.7	33.3

## Data Availability

The raw data supporting the conclusions of this article will be made available by the authors on request.
